# *γδ* T-Cell Acute Lymphoblastic Leukemia/Lymphoma: Discussion of Two Pediatric Cases and Its Distinction from Other Mature *γδ* T-Cell Malignancies

**DOI:** 10.1155/2017/5873015

**Published:** 2017-09-24

**Authors:** Eric X. Wei, Vasiliki Leventaki, John K. Choi, Susana C. Raimondi, Elizabeth M. Azzato, Sheila A. Shurtleff, Menchu G. Ong, Diana M. Veillon, James D. Cotelingam, Rodney E. Shackelford

**Affiliations:** ^1^Department of Pathology and Translational Pathobiology, LSU Health Shreveport, Shreveport, LA, USA; ^2^Department of Pathology, St. Jude Children's Research Hospital, Memphis, TN, USA

## Abstract

Gamma delta (*γδ*) T-cell antigen receptor (TCR) expression and its related T-cell differentiation are not commonly reported in T-cell acute lymphoblastic leukemia/lymphoma (T-ALL). Here we report two pediatric T-ALL cases and present their clinical features, histology, immunophenotypes, cytogenetics, and molecular diagnostic findings. The first patient is a two-year-old girl with leukocytosis, circulating lymphoblasts, and a cryptic insertion of a short-arm segment at 10p12 into the long-arm segment of 11q23 resulting in an MLL and AF10 fusion transcript, which may be the first reported in *γδ* T-ALL. She responded to the chemotherapy protocol poorly and had persistent diseases. Following an allogeneic bone marrow transplant, she went into remission. The second patient is an eleven-year-old boy with a normal white cell count, circulating blasts, and a normal karyotype, but without any immature cellular markers by flow cytometric analysis. He responded to the chemotherapy well and achieved a complete remission. These cases demonstrate the diverse phenotypic, cytogenetic, and molecular aspects of *γδ* T-ALL. Early T-precursor- (ETP-) ALL and their differential diagnosis from other mature *γδ* T-cell leukemia/lymphomas are also discussed.

## 1. Introduction

Gamma delta (*γδ*) T-cell neoplasms are characterized by the expression of the *γδ* T-cell antigen receptors (TCRs), are rare, and include a variety of clinicopathologic entities. T-cell acute lymphoblastic leukemia (T-ALL) with expression of alpha beta (*αβ*) or *γδ* TCR has been previously described in about 35% T-ALL cases, while *γδ* T-ALL cases represented 9–12% of T-ALL including children and adults [1]. Mature T-cell neoplasms with TCR *γδ* expression include hepatosplenic T-cell lymphoma, skin and mucosal *γδ* T-cell lymphoma, and *γδ* T-cell large granular lymphocytic (T-LGL) leukemia [2, 3]. The 2016 revision of the WHO classification of lymphoid neoplasms emphasizes the primary cutaneous *γδ* T-cell lymphoma [4]. *γδ* T-cell lymphomas, other than hepatosplenic T-cell lymphoma and primary cutaneous *γδ* T-cell lymphoma, are often classified within subcategories of T-cell lymphomas. *γδ* T-ALL is uncommon with only 2% of all acute lymphoblastic leukemia (ALL) cases showing expression of the *γδ* TCR [1]. *γδ* TCR expression and T-cell differentiation are not frequently reported in T-ALL [1, 5]. We report clinicopathological features of two pediatric cases of *γδ* T-ALL and discuss the differential diagnosis of other types of *γδ* T-cell leukemia/lymphoma.

## 2. Case Presentation

**Figure 1:**
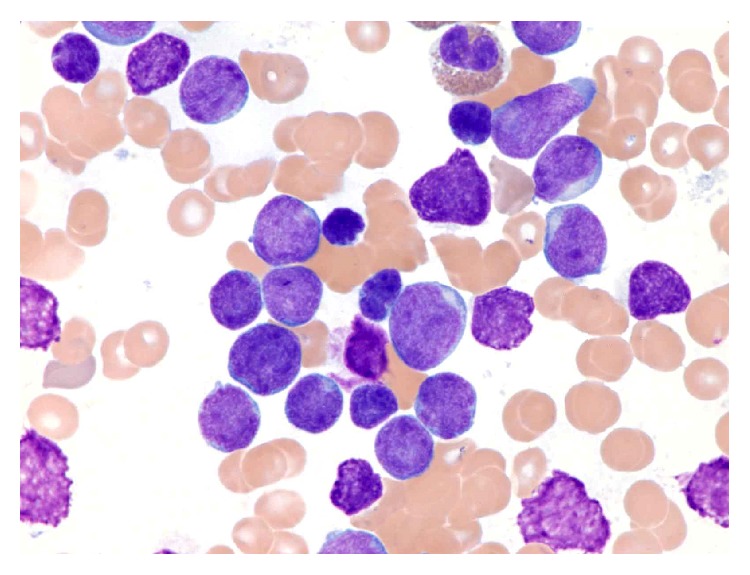
Bone marrow aspirate smear from patient #1, Wright-Giemsa, 1000x.

**Figure 2:**
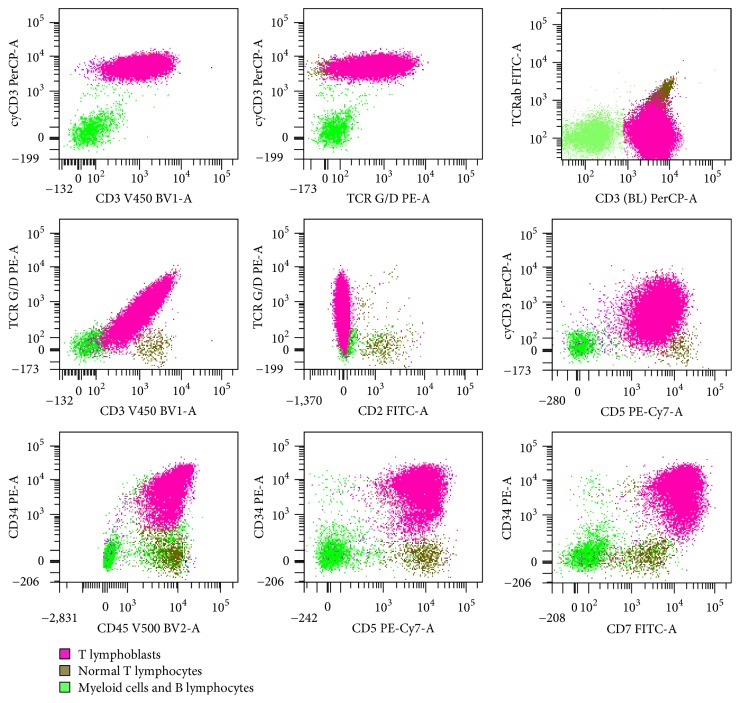
Representative flow cytometric histograms of bone marrow aspirate from patient #1.

**Figure 3:**
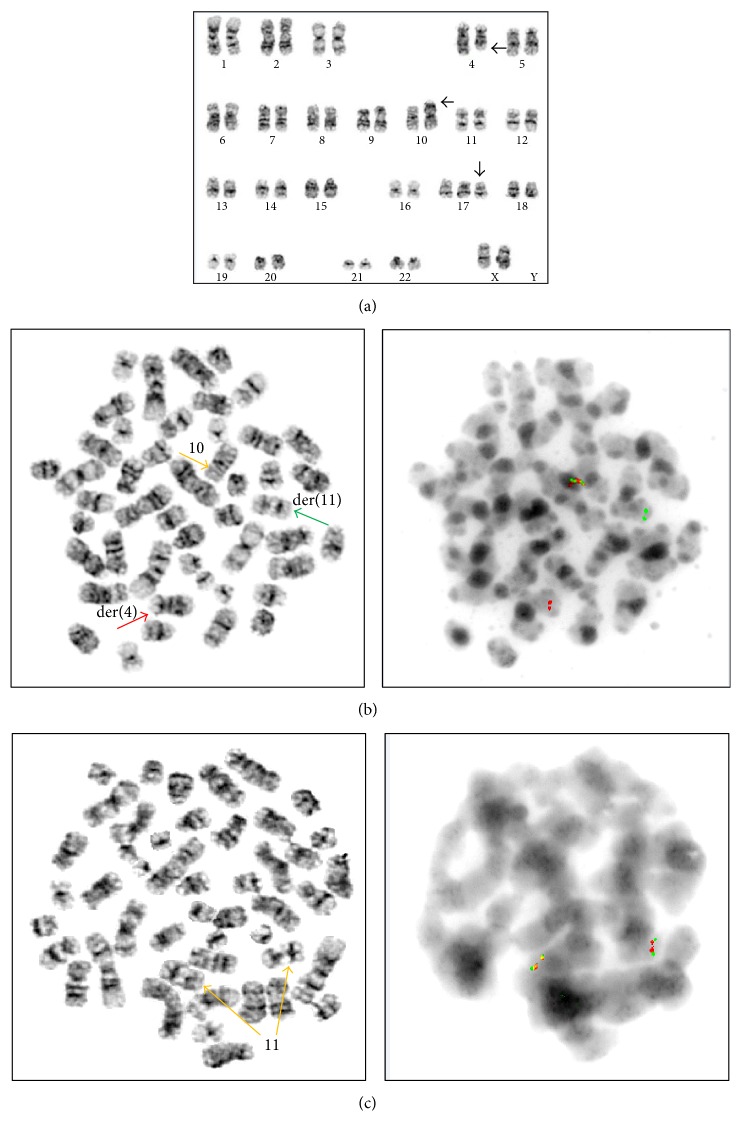
Cytogenetic and FISH results from patient #1. (a) Conventional karyotype with 47, XX, *t*(4;10)(q28;p12), +17. (b) Break-apart AF-10 probe on sequential G-banded to FISH metaphases shows that telomeric 3′ AF10 moves to the 4q28, and the centromeric 5′ AF10 signal inserts into the 11q23 region. (c) The MLL FISH is not rearranged. In (a), the arrows to 4p28 and 10p12 are for indication of *t*(4;10)(q28;p12); the arrow to chromosome 17 is for indication of +17.

**Figure 4:**
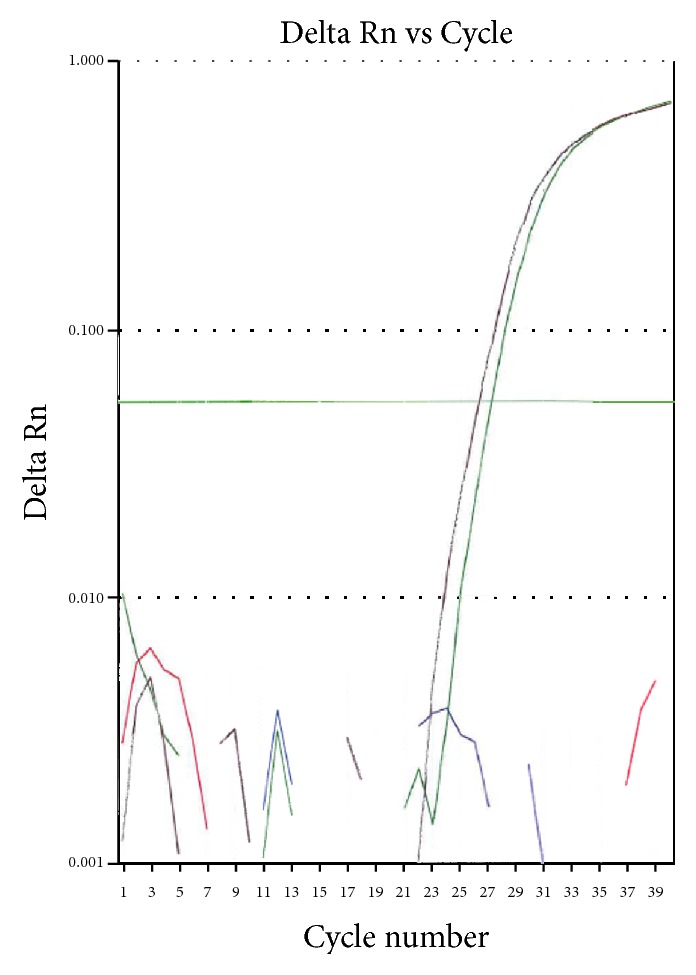
Real-time RT-PCR showing the* MLL/MLLT10* fusion transcripts from patient #1. Delta Rn = normalized fluorescence reporter signal minus baseline; cycle number = cycle of PCR; purple is the positive control; green is the patient; red is no-template control; blue is the negative control (HL60 cell line).

The first case is a 2-year-old Caucasian girl, previously in good health, who suddenly presented at the LSU hospital with fever, shortness of breath, rhinorrhea, cyanosis, and hepatosplenomegaly by chest X-ray. She had anemia and thrombocytopenia, with a white cell count (WBC) at 118.8 thousand/*μ*L and 73% lymphoid cells. Flow cytometry of peripheral blood showed a *γδ* T-cell proliferation. Molecular studies revealed clonal TCR *γ*- and *β*-chain gene rearrangements by PCR. The patient was started on intravenous fluids and antibiotics. She did not undergo a liver or spleen biopsy. Her white cell count decreased to 56.8 thousand/*μ*L the next day and she was transferred to St. Jude Children's Research Hospital (SJCRH). Bone marrow evaluation showed 90% lymphoblasts, exhibiting slightly open chromatin and irregular nuclear contours (Figure 1). Flow cytometry of bone marrow showed T lymphoblasts expressing surface CD3 (variable) and cytoplasmic CD3, CD5, CD7 (bright), CD34, CD45 (dim), and TCR *γδ*. The blasts were negative for CD1a, CD2, CD4, CD8, CD10, CD56, HLA-DR, TCR *αβ*, TdT, and MPO (Figure 2). Cytogenetic studies of the bone marrow showed that 90% of the metaphases had an abnormal karyotype: 47, XX, *t*(4;10)(q28;p12), cryp ins(11;10)(q23;p12p12), +17 (Figure 3(a)). FISH assays using the BCR-ABL1 and TLX3 probes were normal. FISH was also performed using the break-apart AF10 (10p12) probe (research use only) on sequential G-banded to FISH metaphases, and it was found that the probe was rearranged where the telomeric 3′ AF10 moved to the 4q confirming *t*(4;10). Of interest, the centromeric 5′ AF10 signal is inserted into the 11q23 region (Figure 3(b)). By FISH, MLL was not rearranged (Figure 3(c)). The reason MLL was not rearranged or separated because the insertion was very tiny and did not separate enough the 5′ from the 3′ signal. Overall, FISH results indicate a cryptic insertion; that is, a segment of 10p12 had been donated to the recipient 11q23. Molecular studies by real-time RT-PCR assay confirmed an* MLL/MLLT10 (MLL/AF10)* fusion transcript (Figure 4). The patient received treatment per Total XVI (TOTXVI) protocol.

At day 15 following induction, flow cytometry revealed residual disease with 35% blasts in bone marrow. Following reintensification I therapy, the patient had significantly decreased blast percentages but remained persistently positive for minimal residual disease (MRD), with the last MRD before bone marrow transplant (BMT) at 0.018% by flow cytometry. She eventually underwent allogeneic BMT. She had been on day +126 after transplant, remained MRD negative, and had been followed up at the LSU hospital.

**Figure 5:**
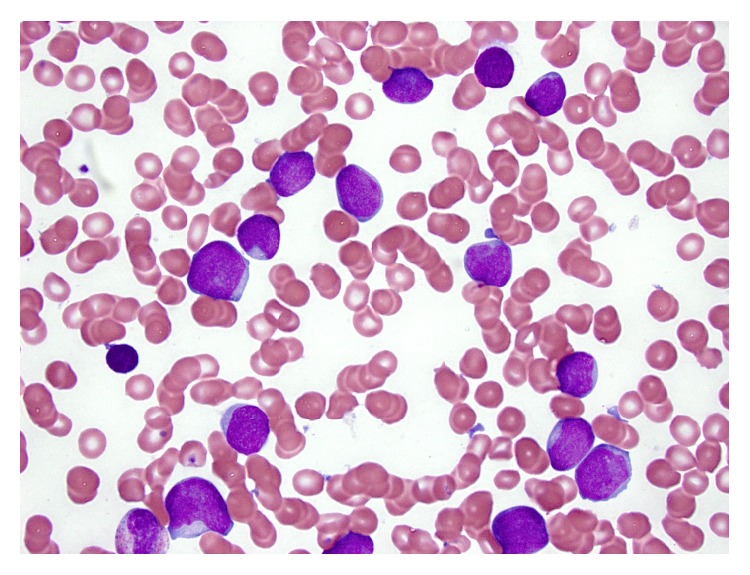
Bone marrow aspirate smear from patient #2, Wright-Giemsa, 1000x.

**Figure 6:**
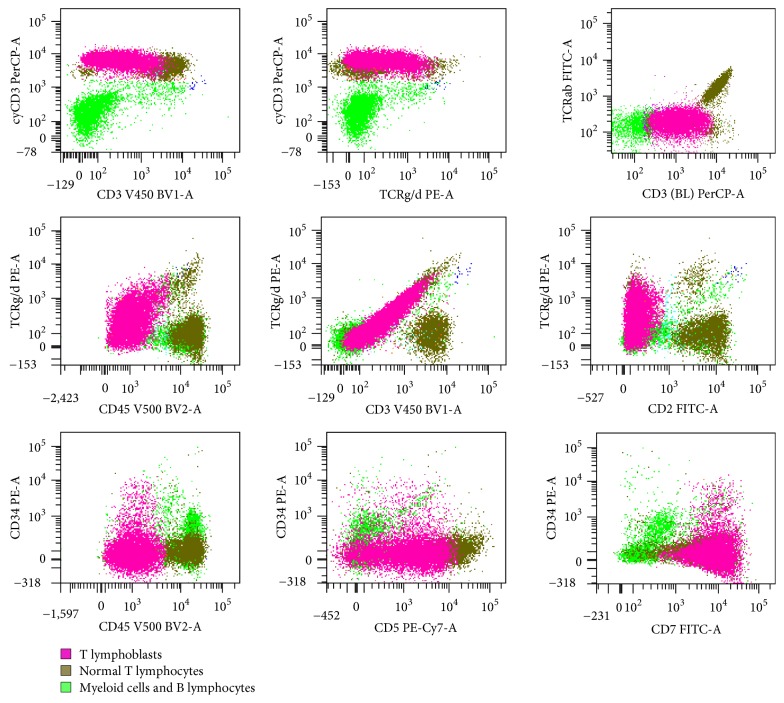
Representative flow cytometric histograms of bone marrow aspirate from patient #2.

The second case is an 11-year-old African American boy with an asthma history who complained of cough, chest tightness, and one-week back pain. His chest X-ray did not show a mediastinal mass. CBC showed a normal white cell count, mild thrombocytopenia, and increased circulating blasts, which appeared to be lymphoblasts morphologically. Flow cytometry revealed approximately 32% circulating *γδ* T-cell lymphoblasts. After maintenance on intravenous fluid and allopurinol therapy, he was transferred to SJCRH for chemotherapy. His bone marrow showed 89% blasts that were small to medium in size with a high nuclear to cytoplasmic ratio, fine chromatin, round to slightly irregular nuclei, and scant cytoplasm (Figure 5). Flow cytometric analysis of the bone marrow confirmed the presence of T lymphoblasts that were positive for surface CD3 (variable), cytoplasmic CD3, CD5 (dim), CD7, CD45 (dim), CD79a, and TCR *γδ*. They were negative for CD1a, CD2, CD4, CD8, CD10, CD34, TdT, MPO, and TCR *αβ* (Figure 6). Cytogenetic studies performed on the bone marrow showed normal a male karyotype without numerical or structural abnormalities. The patient received treatment per Total XVI (TOTXVI) protocol and achieved MRD negativity after induction on day 42. Following remission subsequent to chemotherapy, the patient had been followed up at the LSU hospital.

## 3. Discussion

The differential diagnosis of these two pediatric cases includes skin and mucosal *γδ* T-cell lymphoma in leukemic phase, hepatosplenic T-cell lymphoma, *γδ* T-cell large granular lymphocytic (T-LGL) leukemia, and *γδ* T-ALL [2, 4, 6]. Peripheral *γδ* T-cell lymphoma is a subtype of peripheral T-cell lymphoma, occurring mainly in skin and mucosal regions, often harboring cytotoxic activity. In the skin, *γδ* T-cell lymphoma can be divided into mycosis fungoides-like and primary cutaneous *γδ* T-cell lymphoma, presenting with Sézary syndrome in blood involvement [2, 4, 7]. Mucosal *γδ* T-cell lymphoma may occur in the nasopharynx, lung, gastrointestinal tracts, and other organs, with intestinal *γδ* T-cell lymphoma being type II enteropathy associated [2, 6, 8, 9].

Hepatosplenic T-cell lymphoma is a type of *γδ* T-cell lymphoma with extranodal and systemic involvement [2, 3, 6, 10, 11]. It tends to occur in younger patients with hepatosplenomegaly, systemic symptoms, and cytopenia. The neoplastic cells involve the cords and sinuses of spleen, liver, and bone marrow [12]. The tumor cells are intermediate in size, with condensed chromatin, indistinct nucleoli, and absence of azurophilic granules [11, 12]. Phenotypically, the lymphoma cells are usually positive for CD2, CD3, CD7, CD56, and TCR *γδ* and negative for CD4, CD5, CD8, and TCR *αβ*. Although the first patient showed hepatosplenomegaly with a *γδ* T-cell phenotype, she had marked leukocytosis at the beginning of her disease course, with lymphoblastic appearing neoplastic cells positive for CD5 and CD34 and negative for CD2. Thus, the diagnosis of hepatosplenic T-cell lymphoma is not supported.

In spite of the fact that the majority of T-LGL leukemia cases are of *αβ* type, there are rare cases of *γδ* T-LGL leukemia [2, 13]. In comparison to its *αβ* counterpart, *γδ* T-LGL leukemia patients are prone to having rheumatoid arthritis, lower absolute neutrophil counts, more severe thrombocytopenia, and a higher probability of CD4 and CD8 double negativity. However, both groups of T-LGL leukemia frequently have anemia, an indolent clinical course, and a similar overall survival [2, 13].

T-ALL comprises approximately 20% of all ALL cases [1, 2, 14]. Overall, *γδ* T-ALL is similar to *αβ* T-ALL in the majority of clinical and hematological aspects [4, 14]. T-ALL is more common in children and younger adults, with a male preponderance. The bone marrow is affected in almost all T-ALL cases, and mediastinal or thymic involvement is common. They tend to have a high leukocyte count, lymphadenopathy, and hepatosplenomegaly. Morphologically, the lymphoblasts are intermediate in size and have delicate chromatin, inconspicuous nucleoli, and scant cytoplasm. Immunophenotypically, they are often positive for CD1a, CD2, surface and/or cytoplasmic CD3, CD5, CD7, CD10, CD34, CD45, and TdT but are negative for B cell and myeloid markers. They may be CD4 and CD8 double negative, double positive, or only positive for CD4 or CD8. With cytoplasmic CD3 as the most specific T-cell marker expressed in all maturation stages, there are pro-T (CD7+), pre-T (CD2+ and/or CD5+ and/or CD8+), cortical T (CD1a+), and medullary T (surface CD3+, CD1a−) subtypes of T-ALL based on progressive stages of differentiation [14]. The expression levels of TCR *αβ* or TCR *γδ* in association with different differentiating stages are rarely reported [1]. T-ALL almost always shows clonal TCR gene rearrangements and often carries an abnormal karyotype and an unfavorable prognosis. Early T-precursor- (ETP-) ALL, expressing stem cell or myeloid markers, has a much poorer clinical outcome compared to other T-ALL [14]. Numerous genes have been implicated for the pathogenesis and prognosis for T-ALL, including* NOTCH1*,* TAL1, and HOX1* [5, 14]. *γδ* T-ALL is a rare variant of T lineage lymphoblastic leukemia/lymphoma. Compared to *αβ* T-ALL, *γδ* T-ALL tends to present with lower hemoglobin concentrations in children, more frequent splenomegaly and higher WBC in adults, and higher percentages of the CD45RA−/CD45RO+ phenotype in both children and adults [1]. Although *γδ* T-ALL usually shows TCR gamma and TCR delta chain gene rearrangements, TCR beta chain and biclonal rearrangements involving both V*δ*1 and V*δ*2 segments have also been reported [5]. Both of these two pediatric *γδ* T-ALL patients were likely in medullary (mature) T-ALL stage at presentation with variable surface CD3 expression. Neither of our patients has ETP-ALL due to the strong CD5 expression in the first patient and lack of immature or myeloid markers in the second patient. The cryptic insertion involving MLL and AF10 resulting in the expression of the fusion transcript has rarely been reported in T-ALL [15]. To our knowledge, such cryptic insertion with* MLL/MLLT10 *fusion may be the first reported in *γδ* T-ALL. Previous studies have shown a higher percentage of *γδ* T-ALL, at 9~12%, in comparison with the very low proportion of *γδ* T-cells in the normal thymus, which is at 1% [1]. The reason for this phenomenon is unclear. It is possible that *γδ* T-cells have a higher chance of progression to malignancy compared to *αβ* T-cells or a subset of *γδ* T-ALL may originate from extrathymic tissues [1]. Even though normal *γδ* T-cells are more commonly double negative for CD4 and CD8, a significant percentage of *γδ* T-ALL may exhibit CD4, CD8, or both CD4 and CD8, which may be due to antigen evolution during malignant transformation [1]. *γδ* T-ALL may represent a subcategory of acute lymphoblastic leukemia with slightly distinctive clinical and laboratory features. More studies are needed to further investigate such subtypes of T-ALL, including their pathological diagnosis and clinical management.
